# The gut microbiota-bile acid axis in liver transplantation: implications for postoperative complications and therapeutic strategies

**DOI:** 10.3389/fmicb.2026.1832332

**Published:** 2026-06-19

**Authors:** Churui Xia, Wenjin Zhang, Ten Fang, Longjie Yu, Daijun Yu, Wu Zhang

**Affiliations:** 1 Zhejiang Chinese Medical University, Hangzhou, China; 2 Hangzhou Normal University, Hangzhou, China; 3 Zhejiang University School of Medicine, Hangzhou, China; 4 Zhejiang Shuren University Key Laboratory of Artificial Organs and Computational Medicine, Hangzhou, China; 5 Shulan Hangzhou Hospital Affiliated to Zhejiang Shuren University Shulan International Medical College, Hangzhou, China; 6 Zhejiang Shuren University Shulan International Medical College, Hangzhou, China

**Keywords:** acute rejection, bile acid metabolism, FXR agonist, immunosuppression, ischemia-reperfusion injury, microbiota dysbiosis, postoperative infection, TGR5 agonist

## Abstract

Liver transplantation (LT) is a critical intervention for end-stage liver disease, while complications, such as infections, graft rejection, and metabolic disturbances are common post-transplant. The gut microbiota-bile acid (GM-BA) axis plays a pivotal role in regulating liver function and overall health, influencing both the gut microbiota and bile acid metabolism. This review explored the complex interplay between the gut microbiota (GM) and bile acids during liver transplantation. It also discussed how disruptions in this axis can lead to post-transplant complications, such as infection, rejection, and liver injury. Specifically, the role of microbiota-derived bile acids was assessed in shaping immune responses and metabolic pathways that may impact liver graft function. Furthermore, therapeutic strategies aimed at modulating the GM-BA axis were reviewed to improve post-transplant outcomes, including the use of probiotics, prebiotics, and bile acid receptor modulators. Understanding the mechanisms behind GM-BA dysregulation may provide new directions for improving liver transplant survival and reducing complications.

## 1 Introduction

Liver transplantation (LT) is a life-saving therapy for end-stage liver disease, but postoperative complications, such as hepatic ischemia-reperfusion injury (HIRI), graft dysfunction, rejection, bloodstream infection, biliary infection, multidrug-resistant organism (MDRO) colonization, and metabolic disturbances remain major obstacles to long-term survival (Lucey et al., 2023). Through the portal vein and biliary system, microbial metabolites reach the liver, while bile acids (BAs) shape gut microbial composition and activity.

Under physiological conditions, the products and metabolites of gut microbiota (GM) exert both local and systemic effects to preserve homeostasis of the gut-liver axis (Pabst et al., 2023). As central mediators in this axis, BAs not only play an important role in lipid absorption and excretion, but also act as key regulators in cell signaling pathways, thereby maintaining BA and cholesterol homeostasis, hepatic glucose and lipid metabolism, energy balance, and inflammatory responses. In the enterohepatic circulation, the GM modifies the structure of primary BAs from the liver, affecting their reabsorption efficiency and signaling activity. Conversely, BAs exert regulatory effects on the abundance, diversity and metabolic activity of the GM (Hsu and Schnabl, 2023).

With the resurgence of interest in BA-gut microbiota interactions, the metabolic interplay between these two entities has been extensively characterized in the pathogenesis of liver diseases (Hsu and Schnabl, 2023).

The functional importance of the gut microbiota-bile acid (GM-BA) axis has also been demonstrated in several non-transplant diseases, providing mechanistic insights relevant to LT. In chronic liver disease, dysbiosis-associated alterations in BA metabolism have been shown to influence hepatic stellate cell activation, immune signaling, and the progression of liver fibrosis through farnesoid X receptor (FXR)- and TGR5-mediated pathways. In inflammatory bowel disease, particularly Crohn's disease, emerging evidence indicates that the GM-BA axis regulates mucosal immune responses and metabolic signaling, thereby influencing disease activity and therapeutic responsiveness. For example, altered BA composition and reduced secondary BA-producing bacteria have been associated with impaired immune-metabolic regulation and variable responses to biologic therapies. These findings highlight the broader role of the GM-BA axis in immune and metabolic homeostasis and provide a conceptual framework for understanding how similar mechanisms may influence outcomes after LT (Zhang et al., 2022; Liu et al., 2026).

Patients with end-stage liver disease frequently exhibit pre-transplant gut dysbiosis, characterized by reduced microbial diversity, enrichment of opportunistic pathogens, and altered BA metabolism. Several observational studies suggest that lower microbial alpha diversity before LT is associated with subsequent colonization by MDROs and increased risk of infection after transplantation (Zhang et al., 2022). Following transplantation, however, the GM-BA axis undergoes additional remodeling driven by perioperative factors, including surgical stress, broad-spectrum antibiotic exposure, immunosuppressive therapy, and restoration of graft BA synthesis and bile flow. Consequently, microbiome alterations observed after LT may reflect a combination of baseline disease-related dysbiosis and transplant-related perturbations. In this Review, we explored how LT could modulate the GM-BA axis, whether dysregulation of this axis would be mechanistically associated with post-transplant complications, and the potential for interventional strategies targeting this pathway to improve clinical outcomes after transplantation. To enhance clarity, the main text was organized into thematic sections focusing on post-LT GM-BA remodeling, infection, rejection, ischemia-reperfusion injury, and therapeutic approaches. In this framework, we summarized current evidence regarding GM-BA alterations following LT, with particular emphasis on hepatic ischemia-reperfusion injury, graft dysfunction, infection susceptibility, BA toxicity, macrophage activation, and intestinal barrier injury, as well as emerging therapeutic strategies targeting this axis. Literature for this mini-review was identified through a comprehensive search of the PubMed, Web of Science, and Embase databases for English-language articles published between January 2000 and May 2024. The search utilized combinations of keywords including “liver transplantation,” “gut microbiota,” “microbiome,” “bile acids,” “ischemia-reperfusion injury,” “allograft rejection,” and “immunosuppression.” Priority for inclusion was given to peer-reviewed clinical trials, prospective cohort studies, and high-quality mechanistic animal models that specifically investigated the bidirectional interplay between microbial shifts and BA metabolism in the peri-transplant period. Articles were excluded if they focused solely on pre-transplant liver disease without post-transplant follow-up or lacked relevance to the gut-liver axis.

## 2 Post-transplant remodeling of the GM-BA axis

The remodeling of the GM-BA axis after LT occurs in several temporal phases. Before transplantation, dysbiosis is largely driven by cirrhosis-associated factors, such as portal hypertension, impaired BA synthesis and secretion, intestinal barrier dysfunction, malnutrition, and repeated antibiotic exposure. In the early postoperative period, additional perturbations arise from surgical stress, ischemia-reperfusion injury, intensive care exposure, perioperative antibiotics, and initiation of immunosuppressive therapy. Over longer follow-up periods, partial recovery of microbial diversity may occur as graft function stabilizes and BA metabolism normalizes, although persistent dysbiosis has been reported in some LT recipients. The GM and BA metabolism undergo dynamic yet incomplete remodeling following LT, with implications for clinical outcomes. In high-severity patients, persistent gut dysbiosis is observed post-LT, marked by an overgrowth of pathogens such as *Escherichia coli* and *Shigella flexneri* alongside depletion of butyrate-producing bacteria (*Roseburia intestinalis, Anaerostipes hadrus*). These microbial shifts correlate with hypoalbuminemia and are exacerbated by pre-LT antibiotic exposure and post-LT immunosuppressive agents like tacrolimus (Liu et al., 2026). (Wang et al. 2026) pointed out that postoperative gut dysbiosis involves a phenotypic shift of the gut microbiota from anaerobes toward aerobes and facultative anaerobes. This shift may be driven by an IL-1β-colonic epithelial oxygen metabolism-colonic oxygen environment-gut microbiota regulatory axis. However, these observations are primarily derived from observational clinical studies and demonstrate associations rather than causal relationships between microbiota alterations and post-transplant metabolic changes. Beyond their immunomodulatory functions, commonly used immunosuppressive agents exert direct and indirect effects on the GM-BA axis. Calcineurin inhibitors, such as tacrolimus and cyclosporine, have been associated with reduced microbial diversity and expansion of opportunistic taxa including *Enterococcus* and *Enterobacteriaceae* in observational studies of transplant recipients and experimental models (Zhang et al., 2022). Importantly, much of the mechanistic understanding of these drug–microbiome interactions originates from experimental systems, and direct causal evidence in human liver transplant recipients remains limited. These microbial alterations may also be associated with reduced abundance of BA-transforming bacteria, such as members of *Clostridium* clusters XIVa and IV, which participate in the conversion of primary to secondary Bas (Liu et al., 2026). Experimental studies reported that calcineurin inhibitors can influence hepatic and intestinal BA transport systems, including the bile salt export pump (BSEP), sodium taurocholate cotransporting polypeptide (NTCP), and the apical sodium dependent BA transporter (ASBT), although direct evidence in liver transplant recipients remains limited (Waldner et al., 2023). Other components of standard immunosuppressive regimens may also influence the GM-BA axis. Evidence from experimental studies and non-LT clinical cohorts demonstrates that mycophenolate mofetil can promote gastrointestinal dysbiosis characterized by enrichment of *Proteobacteria* and depletion of short chain fatty acid-producing commensals. Similarly, mTOR inhibitors, such as sirolimus and everolimus may alter microbial metabolic pathways involved in BA transformation, although data specifically in LT recipients remain limited. Therefore, while these findings demonstrate potential interactions between immunosuppressive regimens and the GM-BA axis, the clinical relevance of these mechanisms in human LT populations requires further prospective validation. Corticosteroids can further influence microbial community structure and intestinal barrier function, primarily based on evidence from experimental and non-transplant settings. Collectively, these drug-induced changes may alter the composition of circulating and intestinal BA pools, leading to modified activation of BA receptors, such as FXR and Takeda G protein-coupled receptor 5 (TGR5). Because FXR and TGR5 regulate BA synthesis, epithelial barrier integrity, and innate immune signaling, immunosuppressant-driven perturbations of the GM-BA axis may influence pathways implicated in post-transplant complications. However, direct causal links in human LT recipients remain to be established.

Notably, the timeline of microbial recovery varies across patient subgroups. In children with biliary atresia (BA), LT partially restores gut microbiome diversity within 12–24 months, but significant dysbiosis persists even 2 years post-transplant compared to healthy controls. This delayed recovery is closely linked to impaired synthesis of secondary Bas, recognizing as critical mediators of gut-liver communication (Wang et al., 2026).

**Figure 1:**
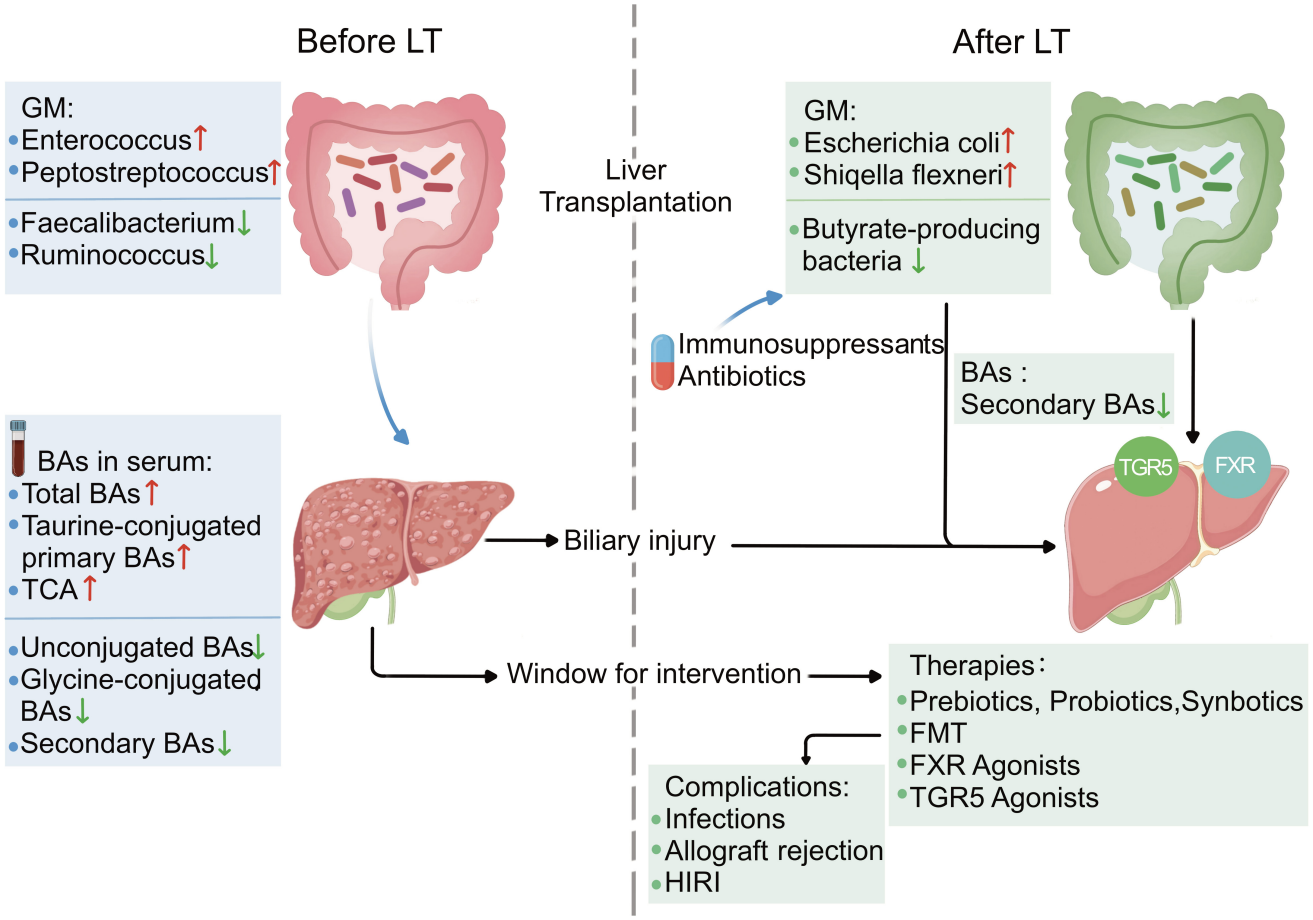
Changes in gut microbiota (GM) and bile acid (BA) metabolism before and after LT. Post-LT dysbiosis is characterized by pathogen expansion and loss of commensals, accompanied by reduced secondary and increased conjugated BAs. Antibiotics and immunosuppressants contribute to these shifts.

Post-LT dysbiosis is further characterized by a decline in commensal bacteria (e.g., *Faecalibacterium prausnitzii, Bacteroides*) and reduced microbial diversity, collectively contributing to complications, such as immune dysregulation, metabolic dysfunction, and gut barrier disruption (Waldner et al., 2023). Nevertheless, most current studies report correlations between microbial composition and clinical outcomes, and the causal contribution of these alterations to post-transplant complications remains to be clarified. (Abenavoli et al. 2023) reviewed the changes in gut microbiota composition before and after LT, hypothesizing possible immune mechanisms linking dysbiosis to transplantation rejection. As shown in Figure 1, LT induces remarkable alterations in gut microbiota composition and BA profiles, characterized by expansion of pathogenic taxa, depletion of commensal microbes, and reduced levels of secondary BAs, changes that are further influenced by antibiotic exposure and immunosuppressive therapy. Importantly, these alterations may also contribute to intestinal barrier disruption, increased bacterial translocation, macrophage activation, and accumulation of potentially hepatotoxic BAs, all of which are implicated in early graft injury and hepatic ischemia-reperfusion injury after LT.

In addition to disease severity and immunosuppressive therapy, several peri-transplant modifiable factors markedly influence early remodeling of the GM-BA axis after LT. Perioperative antibiotic strategies represent one of the strongest determinants of early microbial composition. Broad-spectrum prophylactic antibiotics, which are routinely administered during LT, can markedly reduce microbial diversity and selectively suppress BA-transforming bacteria, such as *Clostridium* and *Ruminococcaceae*, thereby limiting the microbial conversion of primary BAs into immunoregulatory secondary Bas (Hsu and Schnabl, 2023). These antibiotic-induced shifts may favor the expansion of opportunistic pathogens, including *Enterococcus* and *Enterobacteriaceae* and contribute to persistent dysbiosis in the early post-transplant period (Wang et al., 2026). Although such microbiome changes are biologically plausible contributors to post-transplant complications, the majority of evidence linking antibiotic exposure, microbiota disruption, and BA metabolism remains associative. Other perioperative management practices may further shape GM-BA dynamics. Preoperative bowel preparation, although not universally applied in LT, can transiently deplete commensal microbial communities and disrupt short-chain fatty acid-producing taxa, potentially delaying post-transplant microbiome recovery. Nutritional management is another key determinant: early enteral nutrition and fiber-containing diets promote the growth of beneficial commensals and enhance microbial BA transformation, whereas prolonged parenteral nutrition or perioperative fasting may reduce microbial diversity and alter BA signaling pathways. In parallel, enhanced recovery after surgery (ERAS) protocols, characterized by early mobilization, early feeding, and reduced antibiotic exposure, have been associated with improved gut barrier integrity and faster restoration of microbial ecological balance (Waldner et al., 2023). Collectively, these peri-transplant management strategies represent modifiable clinical factors that may influence early GM-BA remodeling and can potentially be optimized to promote microbiome recovery, restore secondary BA production, and improve post-transplant outcomes.

Host genetic variability may represent an additional determinant of inter-individual differences in GM-BA remodeling after LT (Sucu, 2023). Polymorphisms affecting BA transporters and receptors, including the bile salt export pump (BSEP/ABCB11), sodium taurocholate cotransporting polypeptide (NTCP/SLC10A1), and the apical sodium-dependent BA transporter (ASBT/SLC10A2), can influence hepatic BA secretion and enterohepatic circulation (Hsu and Schnabl, 2023; Zhang et al., 2022), thereby shaping the intestinal BA pool that regulates microbial composition. Variants in nuclear receptors that control BA metabolism, such as the FXR/NR1H4 and pregnane X receptor (PXR), may further modify BA synthesis, transport, and downstream signaling pathways involved in metabolic and immune regulation (Waldner et al., 2023). In addition, genetic variants affecting innate immune pathways, including Toll-like receptor 4 (TLR4) and nucleotide-binding oligomerization domain-containing protein 2 (NOD2), may influence mucosal immune responses and susceptibility to infection, thereby indirectly affecting microbial community structure. However, direct evidence regarding the role of host genetic variation in shaping post-transplant GM-BA remodeling in liver transplant recipients remains limited. In addition, host polymorphisms in pattern-recognition receptors, including Toll-like receptors (e.g., TLR4) and nucleotide-binding oligomerization domain-containing proteins (NOD2), can alter host-microbiota interactions and mucosal immune responses (D'Amico et al., 2024), thereby influencing microbial community structure and susceptibility to infection. Collectively, these genetic determinants may contribute to inter-patient variability in post-transplant BA profiles, microbiome recovery, and clinical outcomes, such as cholestasis, infection, and graft dysfunction. However, these hypotheses are largely inferred from studies in non-transplant populations or experimental systems, and their relevance in human LT recipients has not yet been systematically evaluated. Incorporating host genetic factors into future studies of the GM-BA axis may therefore improve risk stratification and enable more personalized microbiome-targeted interventions in LT recipients.

It should be noted that several mechanistic insights into drug-microbiome interactions derive from experimental models or non-LT populations, and causal relationships between immunosuppressive therapy, microbiota alterations, and BA metabolism in LT recipients remain incompletely defined. These findings collectively indicate that LT is associated with remarkable remodeling of the GM-BA axis. While clinical correlations indicate potential implications for graft outcomes, definitive causal relationships require prospective and mechanistic validation (Table 1).

**Table 1:** Gut microbiota and bile acid alterations after liver transplantation.

Aspect	Pre-LT status	Post-LT change	Clinical implication
Microbiota diversity	Reduced in ESLD	Partial recovery, often incomplete	Persistent dysbiosis linked to infection/rejection
Commensals (*Faecalibacterium, Roseburia*)	Depleted	Further reduced by antibiotics and IS drugs	Loss of SCFA production, impaired immune tolerance
Pathogens (*Enterobacteriaceae, Enterococcus, Klebsiella*)	Enriched	Expansion in gut and biliary tract	↑ MDRO infection risk
Secondary bile acids (DCA, LCA, and UDCA)	Low	Remain deficient	↓ Treg induction, impaired colonization resistance
Conjugated bile acids	Elevated	Persistently high	TGR5-mediated immunosuppression, ↑ infection

## 3 The GM-BA axis in post-transplant infections

Increasing evidence demonstrates that alterations in the GM-BA axis are associated with post-transplant infections. Accumulating clinical and experimental evidence further indicates that this axis may influence susceptibility to several distinct infectious complications after LT, including MDRO colonization, bloodstream infection, biliary infection, and invasive fungal infection. Distinct GM features before LT, such as elevated *Klebsiella* and reduced diversity, predispose patients to carbapenem-resistant *Enterobacterales* (CRE) colonization and subsequent infection (D'Amico et al., 2024). It is noteworthy that most studies describing these associations are observational, and whether microbiota alterations directly mediate infection risk or instead reflect underlying disease severity remains uncertain. In parallel, lower levels of fecal metabolites, including short-chain fatty acids and secondary BAs have been associated with increased infection risk, highlighting the metabolic contribution of dysbiosis (Zhang et al., 2022).

### 3.1 MDRO colonization

Several studies have demonstrated that reduced microbial diversity before LT is associated with intestinal colonization by MDROs, particularly carbapenem-resistant *Enterobacterales* and vancomycin-resistant *Enterococcus*. Loss of secondary BA-producing commensals may impair colonization resistance and facilitate pathogen expansion within the intestinal lumen.

### 3.2 Bloodstream infection

Post-LT bloodstream infections are frequently linked to bacterial translocation resulting from intestinal barrier disruption, broad-spectrum antibiotic exposure, and immunosuppressive therapy. Reduced abundance of butyrate-producing bacteria may impair epithelial integrity, while dysregulated BA signaling can alter innate immune responses and macrophage activation.

### 3.3 Biliary infection

Biliary infections and cholangitis may also be influenced by GM-BA alterations. Expansion of *Enterococcus* and *Enterobacteriaceae* in the biliary tract has been associated with altered BA composition and reduced antimicrobial secondary BAs, potentially contributing to biliary inflammation and graft injury.

### 3.4 Fungal infection

In addition to bacterial infections, elevated circulating conjugated BAs and profound dysbiosis may contribute to an immunosuppressed state associated with increased susceptibility to invasive fungal infections, although mechanistic evidence in LT recipients remains limited.

Reduced secondary BA synthesis after LT has been associated with expansion of multidrug-resistant organisms. Experimental studies demonstrate that secondary BAs can promote colonization resistance, but whether this mechanism directly mediates infection risk in LT recipients remains to be confirmed (Wirth et al., 2023). Elevated circulating levels of conjugated BAs may suppress innate immune responses through activation of TGR5 signaling, contributing to an immunosuppressive or “immunoparalyzed” state that is associated with an increased risk of bacterial and fungal infections (Leonhardt et al., 2023). Moreover, mucosal-associated invariant T (MAIT) cells, which are innate-like lymphocytes enriched in the liver and intestinal mucosa, are significantly depleted after LT, further weakening host antimicrobial defense (Wang W. et al., 2025). Collectively, these findings demonstrate that GM-BA alterations may influence infection susceptibility, although most clinical data remain associative.

## 4 The GM-BA axis in allograft rejection

Liver transplant recipients experiencing acute rejection consistently exhibit reduced microbial diversity and marked compositional shifts, characterized by the expansion of potentially pathogenic taxa, such as *Bacteroides* and *Enterobacteriaceae*, alongside depletion of beneficial commensals including *Ruminococcaceae* and *Lactobacillaceae*. These changes are often accompanied by sustained reductions in *Faecalibacterium*, which have been associated with decreased regulatory T cell (Treg) populations and enhanced Th17 responses, reflecting a pro-inflammatory immune state (Barbetta et al., 2024). In addition, several gut-derived metabolites play critical roles in modulating immune homeostasis following LT. Short-chain fatty acids and secondary BAs promote Treg differentiation while inhibiting Th17 responses, whereas commensals such as *Bacteroides fragilis, Bifidobacterium*, and *Akkermansia muciniphila* enhance anti-inflammatory cytokine production, collectively supporting graft tolerance (Lee et al., 2025). Preclinical studies demonstrate that microbial metabolites influence the Treg/Th17 balance. In LT recipients, reduced microbial diversity has been associated with acute rejection, while causality has not been established.

Bile composition has emerged as a diagnostic and prognostic marker in LT. During acute rejection, biliary cytokines and miRNAs rise earlier and more specifically than serum markers, while altered bile salt/phospholipid ratios and elevated conjugated BAs correlate with rejection and biliary injury. Beyond serving as biomarkers, secondary BAs have shown in experimental models to modulate immune cell differentiation and function (Tao et al., 2025). Whether these mechanisms operate similarly in human LT recipients requires further study.

In addition to recipient-derived microbiota, recent studies have demonstrated that donor livers may harbor distinct microbial DNA signatures detectable in organ preservation solution (OPS). Lucas-Ruiz et al. reported that these intrahepatic microbial profiles were associated with acute rejection, vascular and biliary complications, and patient survival. Machine-learning models built on OPS microbial features showed high predictive accuracy, indicating that OPS-based microbiome assessment may serve as a potential non-invasive approach for graft risk stratification (Lucas-Ruiz et al., 2025). While these findings are promising, they are based on associative modeling and require independent validation before clinical implementation.

## 5 The GM-BA axis in HIRI

Evidence linking the GM-BA axis to HIRI derives primarily from experimental animal models, with limited human corroboration. HIRI is a major contributor to early graft dysfunction and rejection, accounting for up to 10% of early LT failures (Wang H. et al., 2025). Increasing evidence indicates that gut microbiota and its metabolites exert protective roles against HIRI. In murine models, microbial-derived 3,4-dihydroxyphenylpropionic acid (3,4-DHPPA) attenuates macrophage-mediated inflammation via HDAC inhibition, while glutamine-derived α-ketoglutarate promotes M2 macrophage polarization and mitigates liver injury (Lu et al., 2023). Similarly, isoursodeoxycholic acid (IsoUDCA), a secondary BA, has shown in mouse models to protect against HIRI by engaging the nuclear receptor Nur77 and suppressing NF-κB signaling (Li et al., 2025). Mechanistically, disruption of the intestinal barrier during LT may promote translocation of microbial products, such as lipopolysaccharide into the portal circulation, thereby enhancing Kupffer cell and macrophage activation within the graft. Concurrent reductions in protective secondary BAs and accumulation of potentially hepatotoxic BAs may further amplify inflammatory signaling, oxidative stress, and hepatocellular injury during reperfusion.

Limited clinical studies report correlations between preoperative microbial signatures and biochemical markers of reperfusion injury; however, mechanistic confirmation in humans is lacking. In LT recipients, a higher preoperative abundance of *Lachnospiraceae* has been reported to be associated with lower levels of liver enzymes and inflammatory cytokines following reperfusion, being consistent with animal studies showing that *Lachnospiraceae* improves intestinal barrier integrity and attenuates ferroptosis (Deng et al., 2025; Wang F. et al., 2024).

Together, these findings highlight the GM-BA axis as a key modulator of HIRI pathogenesis, linking microbial metabolites, immune regulation, and systemic consequences.

## 6 Therapeutic strategies targeting the GM-BA axis

### 6.1 Modulation of gut microbiota

Several therapeutic strategies have been proposed to restore microbiota balance and regulate BA signaling after LT (Table 2). Prebiotics, probiotics, and synbiotics are widely available and commonly used to modulate the gut microbiota. These interventions have been reported to ameliorate microbial dysbiosis, strengthen gut barrier integrity, and modulate immune responses by promoting the production of beneficial metabolites, such as short-chain fatty acids. A recent systematic review and meta-analysis of 12 studies, including five involving LT recipients, showed that perioperative administration of probiotics or synbiotics significantly reduced postoperative infections, length of hospital stay, and antibiotic use, highlighting their potential clinical value in transplant care (Wu et al., 2025). Consistent with these findings, a meta-analysis by Karitnig et al., which included 19 randomized controlled trials (eight involving LT), demonstrated that perioperative probiotic or synbiotic supplementation significantly reduced postoperative infectious complications (OR = 0.34, 95% CI 0.25–0.45), shortened hospital stay, and improved indicators of systemic inflammation and liver function (e.g., serum endotoxin, white blood cell count, ALT, AST, bilirubin, and INR; Karitnig et al., 2025). Although these trials demonstrate reduced infection rates, most studies did not directly assess BA profiles, and mechanistic pathways remain incompletely characterized. Despite these promising findings, several limitations currently restrict the routine clinical implementation of microbiota-targeted therapies in liver transplant recipients. Probiotic and synbiotic formulations vary substantially across studies with respect to microbial strains, dosing regimens, and treatment duration, making direct comparison and standardization challenging. In addition, microbiome-targeted therapeutic protocols have not yet been standardized for transplant populations. Importantly, long-term safety data remain limited, particularly in immunosuppressed patients, in whom theoretical risks include opportunistic infection or unintended microbial translocation.

**Table 2:** Therapeutic strategies targeting the GM-BA axis in liver transplantation.

Strategy	Mechanism	Evidence in LT	Limitations
Probiotics/synbiotics	Restore commensals, enhance barrier, ↑ SCFAs	↓ Infections and hospital stay (meta-analysis of 12 RCTs, incl. LT)	Strain-specific effect, heterogeneity
FMT	Recolonization with donor microbiota	Effective for recurrent CDI in LT patients	Risk of viral/bacterial transmission
FXR agonists (OCA, C7)	Regulate BA homeostasis, ↓ inflammation	Preclinical + early clinical studies	Pruritus, endothelial FXR paradox
TGR5 agonists (INT-777, derivatives)	Anti-inflammatory, metabolic benefits	Preclinical promise	Safety concerns (GB filling)
β-glucan (Lentinan)	↑ IsoUDCA, suppress macrophage inflammation	Animal models (HIRI)	Lacking clinical validation

Experimental studies further support microbiota-targeted approaches. In murine models, oral administration of lentinan, a β-glucan polysaccharide, alleviated hepatic ischemia-reperfusion injury by reshaping the gut microbiota and increasing microbiota-derived isoursodeoxycholic acid (IsoUDCA). IsoUDCA in turn suppressed macrophage inflammation via Nur77 activation, illustrating a novel microbiota-BA-immune signaling axis (Wang F. et al., 2024).

Fecal microbiota transplantation (FMT) has also shown promise in solid organ transplant recipients, particularly for recurrent *Clostridioides* difficile infection. While generally safe and effective, FMT requires careful patient selection and monitoring due to risks of infection, viral reactivation, or rejection.

### 6.2 BA receptor modulators

BA receptors represent promising therapeutic targets in LT. FXR, highly expressed in hepatocytes and enterocytes, regulates BA and metabolic homeostasis and exerts anti-inflammatory effects via intestinal and hepatic signaling pathways (Song et al., 2025). Obeticholic acid (OCA), the first FXR agonist in clinical use, demonstrated efficacy but was limited by pruritus through off-target activation of MRGPRX4. A novel derivative, C7, retains FXR agonism without inducing itch in preclinical studies (Yang et al., 2024; Wang K. et al., 2024). Although FXR activation generally protects against cholestasis, recent evidence suggests context-dependent effects, as endothelial FXR signaling may promote neutrophil recruitment and exacerbate inflammation (Zhang et al., 2025).

**Figure 2:**
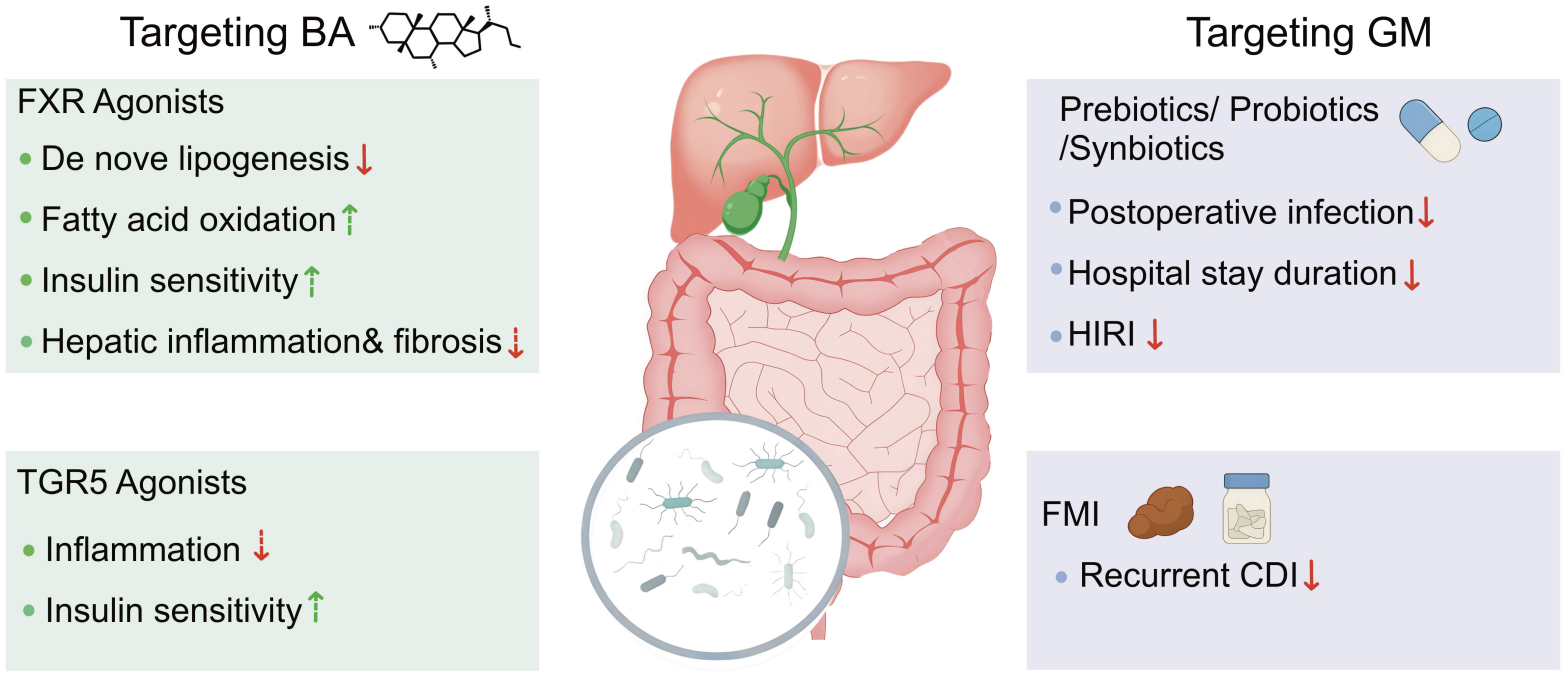
Therapeutic strategies targeting the GM-BA axis in liver transplantation. Microbiota-directed approaches (probiotics, prebiotics, FMT) improve barrier function and reduce infection. BA receptor agonists (FXR, TGR5) modulate inflammation and metabolism. Together, these interventions aim to lower risks of infection, rejection, and ischemia-reperfusion injury.

The G protein-coupled receptor TGR5 mediates anti-inflammatory and metabolic effects, making it another attractive target. However, no TGR5 agonist has achieved clinical approval due to safety concerns such as gallbladder filling. Compounds including INT-777 and gut-restricted derivatives show preclinical promise but remain under investigation (Jin et al., 2024). Furthermore, translation of BA receptor-targeted therapies into routine clinical practice faces several challenges, including limited transplant-specific clinical trials, uncertainty regarding optimal dosing strategies, and incomplete understanding of long-term safety in immunosuppressed populations. Most data on FXR and TGR5 agonists in the transplant setting were derived from preclinical or non-transplant populations. Human studies are largely observational and demonstrate associations between microbial composition, BA metabolism, and post-transplant complications. Mechanistic insights are primarily derived from experimental animal models, while interventional evidence remains limited to microbiota directed therapies. Representative human and experimental studies examining the GM-BA axis in LT are summarized in Table 3, highlighting that most clinical evidence remains associative, whereas mechanistic insights largely derive from animal models. A schematic overview of these therapeutic strategies, including microbiota-directed interventions and BA receptor agonists, is presented in Figure 2.

**Table 3:** Representative human and experimental studies on the GM-BA axis in liver transplantation.

Study	Study design/model	Key findings related to GM-BA axis	Relevance to LT outcomes
(Lehmann et al. 2024)	Human LT recipients; fecal metabolomics	Distinct microbial metabolite profiles, including bile acid-related metabolites, identified in patients at risk for postoperative infection	Metabolite signatures predicted infection risk after LT
(Vega-Abellaneda et al. 2024)	Prospective human LT cohort	Gut microbiome composition partially restored after transplantation; recovery influenced by antibiotics and underlying disease	Microbiome recovery associated with post-transplant clinical course
(Waldner et al. 2023)	Pediatric biliary atresia patients undergoing LT	LT reshaped gut microbiota and altered bile acid homeostasis	Demonstrated microbiome-bile acid interplay after LT
(D'Amico et al. 2024)	Prospective observational LT study	Dysbiosis characterized by *Enterobacterales* expansion and altered microbial metabolism	Microbiome dynamics associated with post-transplant infections
(Lu et al. 2023)	Mouse model of hepatic ischemia-reperfusion injury	Microbiota-derived glutamine regulated macrophage metabolic reprogramming	Reduced liver ischemia-reperfusion injury relevant to transplant graft injury
(Li et al. 2025)	Mouse model	Microbiota modulation increased isoursodeoxycholic acid (isoUDCA) levels	Elevated bile acid metabolite protected against hepatic IRI
(Deng et al. 2025)	Steatotic donor liver experimental model	*Lachnospiraceae* species modulated oxidative stress and ferroptosis pathways	Reduced ischemia-reperfusion injury in donor livers
(Wang F. et al. 2024)	Mouse model	Gut microbiota-derived GABA inhibited ferroptosis during liver injury	Attenuated ischemia-reperfusion injury relevant to transplantation

## 7 Conclusions and perspectives

Clinical outcomes following LT are increasingly recognized to be influenced by the GM-BA axis; however, the underlying mechanistic basis remains incompletely defined. Although numerous studies have characterized alterations in gut microbial composition before and after LT, relatively few have directly connected these changes to BA metabolism or graft physiology. A clearer delineation of these interactions is essential for the development of novel strategies aimed at improving long-term graft survival.

Therapeutic options targeting this axis are beginning to emerge. Pharmacological agonists of the FXR and the TGR5 have demonstrated promising effects in preclinical models and early-phase clinical studies. In parallel, microbiota-directed interventions, including probiotics and prebiotics, have shown potential benefits in reducing post-transplant infectious complications (Anila et al., 2025).

Future investigations should address several key priorities. Mechanistic studies employing gnotobiotic transplantation models, intestinal and hepatic organoid systems, and integrated multi-omics approaches will be critical for establishing causal relationships between microbial alterations and BA-mediated signaling pathways. In addition, precision modulation of the gut microbiota, such as individualized probiotic or synbiotic strategies, may promote restoration of secondary BA-producing capacity and immunological tolerance. Longitudinal profiling of BA composition may also provide non-invasive biomarkers of graft status, enabling discrimination between protective and pro-inflammatory BA signatures. Finally, well-designed, transplant-specific clinical trials are required to accurately evaluate microbiota- and BA-targeted therapeutic strategies in liver transplant recipients.

In conclusion, advancing mechanistic insight into the GM-BA axis in LT represents an important research priority. Integrative therapeutic approaches that incorporate microbial, metabolic, and BA-directed strategies may ultimately contribute to the reduction of postoperative complications and the improvement of long-term graft and patient outcomes.

## References

[B1] Abenavoli L. Scarlata G. G. M. Paravati M. R. Boccuto L. Luzza F. Scarpellini E. . (2023). Gut microbiota and liver transplantation: immune mechanisms behind the rejection. Biomedicines 11:1792. doi: 10.3390/biomedicines11071792 37509432 PMC10376769

[B2] Anila K. N. Nair S. S. Balakrishnan D. Unnikrishnan G. Binoj S. T. Menon R. N. . (2025). FXR agonist in post-liver transplantation patients: a randomized open-labeled study. F1000Research 14:9. doi: 10.1016/j.jceh.2024.102189 PMC13560005 42728928

[B3] Barbetta A. Rocque B. Bangerth S. Street K. Weaver C. Chopra S. . (2024). Spatially resolved immune exhaustion within the alloreactive microenvironment predicts liver transplant rejection. Sci Adv. 10:eadm8841. doi: 10.1126/sciadv.adm8841 38608023 PMC11014454

[B4] D'Amico F. Rinaldi M. Pascale R. Fabbrini M. Morelli M. C. Siniscalchi A. . (2024). Gut microbiome dynamics and *Enterobacterales* infection in liver transplant recipients: a prospective observational study. JHEP Rep. 6:101039. doi: 10.1016/j.jhepr.2024.101039 38524669 PMC10960129

[B5] Deng S. Cao H. Li T. Wang X. Meng J. Zeng T. . (2025). *Lachnospiraceae*-bacterium alleviates ischemia-reperfusion injury in steatotic donor liver by inhibiting ferroptosis via the Foxo3-Alox15 signaling pathway. Gut Microbes 17:2460543. doi: 10.1080/19490976.2025.2460543 39882747 PMC11784649

[B6] Hsu C. Schnabl B. (2023). The gut-liver axis and gut microbiota in health and liver disease. Nat. Rev. Microbiol. 21, 719–733. doi: 10.1038/s41579-023-00904-3 37316582 PMC10794111

[B7] Jin W. Zheng M. Chen Y. Xiong H. (2024). Update on the development of TGR5 agonists for human diseases. Eur. J. Med. Chem. 271:116462. doi: 10.1016/j.ejmech.2024.116462 38691888

[B8] Karitnig R. Bogner A. Jahn N. Vlachos C. Lederer A. Geisler A. . (2025). Value of probiotics on outcome in patients following liver surgery: a systematic review and meta-analysis. Medicina (Kaunas) 61:1068. doi: 10.3390/medicina61061068 40572756 PMC12194926

[B9] Lee S. K. Kwon J. H. Jang J. W. Bae S. H. Yoon S. K. Jung E. S. . (2025). The critical role of regulatory T cells in immune tolerance and rejection following liver transplantation: interactions with the gut microbiome. Transplantation 109, 784–793. doi: 10.1097/TP.0000000000005220 39375899

[B10] Lehmann C. J. Dylla N. P. Odenwald M. Nayak R. Khalid M. Boissiere J. . (2024). Fecal metabolite profiling identifies liver transplant recipients at risk for postoperative infection. Cell Host Microbe. 32, 117–130.e4. doi: 10.1016/j.chom.2023.11.016 38103544

[B11] Leonhardt J. Dorresteijn M. J. Neugebauer S. Mihaylov D. Kunze J. Rubio I. . (2023). Immunosuppressive effects of circulating bile acids in human endotoxemia and septic shock: patients with liver failure are at risk. Crit. Care 27:372. doi: 10.1186/s13054-023-04620-5 37759239 PMC10523742

[B12] Li J. Bao J. Liu Y. Chen M. Chen Y. Tuolihong L. . (2025). Lentinan enhances microbiota-derived isoursodeoxycholic acid levels to alleviate hepatic ischemia-reperfusion injury in mice. Int. J. Biol. Macromol. 304(Pt 2):140717. doi: 10.1016/j.ijbiomac.2025.140717 39920949

[B13] Liu L. Liang L. Liang H. Wang M. Zhou W. Mai G. . (2026). Microbiome-metabolome generated bile acids gatekeep infliximab efficacy in Crohn's disease by licensing M1 suppression and Treg dominance. J. Adv. Res. 83, 789–806. doi: 10.1016/j.jare.2025.08.017 40812589 PMC13131408

[B14] Lu T. Li Q. Lin W. Zhao X. Li F. Ji J. . (2023). Gut microbiota-derived glutamine attenuates liver ischemia/reperfusion injury via macrophage metabolic reprogramming. Cell. Mol. Gastroenterol. Hepatol. 15:1255. doi: 10.1016/j.jcmgh.2023.01.004 36706918 PMC10140379

[B15] Lucas-Ruiz F. Vidal-Correoso D. Mateo S. V. de la Torre-Álamo M. M. Jover-Aguilar M. Alconchel F. . (2025). Intrahepatic donor microbiota-based metataxonomic signature detected in organ preservation solution enables prediction of short-term liver transplant outcomes. Gut 74, 2058–2069. doi: 10.1136/gutjnl-2025-335986 40803752 PMC12703288

[B16] Lucey M. R. Furuya K. N. Foley D. P. (2023). Liver transplantation. N. Engl. J. Med. 389, 1888–1900. doi: 10.1056/NEJMra2200923 37966287

[B17] Pabst O. Hornef M. W. Schaap F. G. Cerovic V. Clavel T. Bruns T. . (2023). Gut-liver axis: barriers and functional circuits. Nat. Rev. Gastroenterol. Hepatol. 20, 447–461. doi: 10.1038/s41575-023-00771-6 37085614

[B18] Song L. Hou Y. Xu D. Dai X. Luo J. Liu Y. . (2025). Hepatic FXR-FGF4 is required for bile acid homeostasis via an FGFR4-LRH-1 signal node under cholestatic stress. Cell Metab. 37, 104.e9–120.e9. doi: 10.1016/j.cmet.2024.09.008 39393353

[B19] Sucu S. (2023). Impact of gut microbiota on liver transplantation. Am. J. Transpl. 23, 1485–1495. doi: 10.1016/j.ajt.2023.05.030 37277064

[B20] Tao Z. Luo Z. Zou Z. Ye W. Hao Y. Li X. . (2025). Novel insights and an updated review of metabolic syndrome in immune-mediated organ transplant rejection. Front Immunol. 16:1580369. doi: 10.3389/fimmu.2025.1580369 40330480 PMC12052740

[B21] Vega-Abellaneda S. Dopazo C. Ya1ez F. Soler Z. Xie Z. Canalda-Baltrons A. . (2024). Microbiome composition recovery after liver transplantation correlates with initial liver disease severity and antibiotics treatment. Am. J. Transplant. 24, 1623–1633. doi: 10.1016/j.ajt.2024.03.038 38556088

[B22] Waldner B. Aldrian D. Zöggeler T. Oberacher H. Oberhuber R. Schneeberger S. . (2023). The influence of liver transplantation on the interplay between gut microbiome and bile acid homeostasis in children with biliary atresia. Hepatol. Commun. 7:e0151. doi: 10.1097/HC9.0000000000000151 37184522 PMC10187839

[B23] Wang F. Liu X. Huang F. Zhou Y. Wang X. Song Z. . (2024). Gut microbiota-derived gamma-aminobutyric acid from metformin treatment reduces hepatic ischemia/reperfusion injury through inhibiting ferroptosis. eLife 12:RP89045. doi: 10.7554/eLife.89045.4 38488837 PMC10942780

[B24] Wang H. Guo M. Ren B. Zhang H. Zhang J. Qiao R. . (2025). Circadian control of hepatic ischemia/reperfusion injury via HSD17B13-mediated autophagy in hepatocytes. J. Hepatol. 83, 750–767. doi: 10.1016/j.jhep.2025.02.029 40049242

[B25] Wang K. Zhang Y. Wang G. Hao H. Wang H. (2024). FXR agonists for MASH therapy: lessons and perspectives from obeticholic acid. Med. Res. Rev. 44, 568–586. doi: 10.1002/med.21991 37899676

[B26] Wang W. Dai C. Zhu P. Wu M. Zhang H. Wei Q. . (2025). Liver transplant-facilitated CD161+Vα7.2+ MAIT cell recovery demonstrates clinical benefits in hepatic failure patients. Nat Commun. 16:4022. doi: 10.1038/s41467-025-59308-x 40301342 PMC12041255

[B27] Wang W. Liao X. Liu J. Liu B. Zhou C. Liang P. . (2026). Elevated postoperative IL-1β induces disorder of intestinal microenvironment and alteration of gut microbiota. Front. Microbiol. 17:1744636. doi: 10.3389/fmicb.2026.1744636 41948039 PMC13050866

[B28] Wirth U. Jiang T. Schardey J. Kratz K. Li M. Schirren M. . (2023). The role of microbiota in liver transplantation and liver transplantation-related biliary complications. Int. J. Mol. Sci. 24:4841. doi: 10.3390/ijms24054841 36902269 PMC10003075

[B29] Wu H. Guan Z. Zhang K. Zhou L. Cao L. Mou X. . (2025). The effect of perioperative probiotics and synbiotics on postoperative infections in patients undergoing major liver surgery: a meta-analysis of randomized controlled trials. PeerJ 13:e18874. doi: 10.7717/peerj.18874 39981042 PMC11841616

[B30] Yang J. Zhao T. Fan J. Zou H. Lan G. Guo F. . (2024). Structure-guided discovery of bile acid derivatives for treating liver diseases without causing itch. Cell 187, 7164.e18–7182.e18. doi: 10.1016/j.cell.2024.10.001 39476841

[B31] Zhang P. Li X. Liang J. Zheng Y. Tong Y. Shen J. . (2025). Chenodeoxycholic acid modulates cholestatic niche through FXR/Myc/P-selectin axis in liver endothelial cells. Nat. Commun. 16:2093. doi: 10.1038/s41467-025-57351-2 40025016 PMC11873286

[B32] Zhang Y. L. Li Z. J. Gou H. Z. Song X. J. Zhang L. (2022). The gut microbiota-bile acid axis: a potential therapeutic target for liver fibrosis. Front. Cell Infect. Microbiol. 12:945368. doi: 10.3389/fcimb.2022.945368 36189347 PMC9519863

